# EVALUATION OF THE KNOWLEDGE ON COST OF ORTHOPEDIC IMPLANTS AMONG ORTHOPEDIC SURGEONS

**DOI:** 10.1590/1413-785220162404153822

**Published:** 2016

**Authors:** Gustavo Gonçalves Arliani, Rodrigo Guerra Sabongi, Alysson Ferreira Batista, Diego Costa Astur, Guilherme Guadagnini Falotico, Moises Cohen

**Affiliations:** 1. Universidade Federal de São Paulo, Department of Orthopedics and Traumatology, Sports Traumatology Center, São Paulo, SP, Brazil.

**Keywords:** Prosthesis implantation, Cost control, Cost savings, Hospital costs, Orthopedics

## Abstract

**Objective:**

To determine the knowledge of Brazilian Orthopedic Surgeons on the costs of orthopedic surgical devices used in surgical implants***.***

**Methods:**

A questionnaire was applied to Brazilian Orthopedic Surgeons during the 46^th^ Brazilian Congress on Orthopedics and Traumatology***.***

**Results:**

Two hundred and one Orthopedic Surgeons completely filled out the questionnaire. The difference between the average prices estimated by the surgeons and the average prices provided by the supplier companies was 47.1%. No differences were found between the orthopedic specialists and other subspecialties on the prices indicated for specific orthopedic implants. However, differences were found among orthopedic surgeons who received visits from representatives of implant companies and those who did not receive those visits on prices indicated for shaver and radiofrequency device. Correlation was found between length of orthopedic experience and prices indicated for shaver and interference screw, and higher the experience time the lower the price indicated by Surgeons for these materials***.***

**Conclusion:**

The knowledge of Brazilian Orthopedic Surgeons on the costs of orthopedic implants is precarious. Reduction of cost of orthopedics materials depends on a more effective communication and interaction between doctors, hospitals and supplier companies with solid orientation programs and awareness for physicians about their importance in this scenario.***Level of Evidence III, Cross-Sectional Study.***

## INTRODUCTION

Orthopaedics and Traumatology is the specialty of medicine responsible for the diagnosis and treatment of trauma to the skeletal muscles of the human body structures, as well as other disorders of the locomotor system. The word Orthopedics comes from the Greek, where "*ortho*" means straight, right and "*pedics*", child.

The enormous importance of the specialty comes from the pain-relieving capacity, restoration of function and correction of patients' deformities through the various existing orthopedic treatments.

In recent decades there have been significant advances in the area with the advent of increasingly less invasive techniques for patients. These changes are only possible due to the appearance, innovation and improvement of the surgical orthopedics materials sector.^1^ However, these new technologies may be the most responsible for the abrupt and constant increasing costs in healthcare. For these expenses to be controlled and better managed it is important that administrators, nurses, health insurance plans and particularly physicians have complete science of the amounts involved in health procedures.^2^

Previous studies, however, showed that orthopedic surgeons often do not have an exact idea of ​​the values ​​of orthopedics materials used in surgeries.^3^ ^-^ ^5^ Several studies have been conducted in recent years assessing the prospects and trends of Brazilian orthopedic surgeons. But all these studies had as main theme orthopedic conditions and did not address health management and costs.^6^ ^-^ ^8^

Therefore, the aim of this study is to determine the knowledge of Brazilian orthopedic surgeons on the cost of implants used in many orthopedic surgical procedures.

## METHODS

This is a descriptive study using a questionnaire to a sample of orthopedic surgeons in Brazil. The questionnaire was prepared and approved by the authors so that it was very understanding and simple. It consisted of questions covering topics such as practice time, number of surgeries per year performed in the public and private sectors, subspecialty of the surgeons and estimate price of many orthopedics materials. (Annex 1)

The questionnaire was applied to Brazilian orthopedic surgeons during the three days of the 46^th^ Brazilian Congress of Orthopedics and Traumatology. To resolve any questions while filling it, one of the authors of this paper was always present throughout the application period of the questionnaires. The prices of several orthopedics materials were requested for three different companies and an average price was obtained.

From the data from the questionnaires, a demographic descriptive statistics of the variables involved to characterize the sample was conducted. In order to perform a correlation between the orthopedics subspecialty within orthopedics and the prices indicated for orthopedics materials and a correlation between receiving visits from representatives of suppliers companies and the price of the materials, we used the Mann-Whitney test. In the correlation analysis between prices of materials (R$) and experience time of orthopedists we used the Spearman correlation coefficient. Data were analyzed using SPSS for Windows version 16.0 and a significance level of 5% was adopted. The study was approved by the Research Ethics Committee of *Universidade Federal de São Paulo*, São Paulo, SP, Brazil, under number 1.283.422.

## RESULTS

In total, 201 orthopedic surgeons completely filled out the questionnaire and were part of the sample analyzed. Table 1 shows the geographic distribution of surgeons according to the region of origin. Regarding the surgeons' time of experience, we obtained an average of 7.4 ± 8.8 years (range 1-40 years). The results on the number of surgeries performed per year in the public and private sectors are shown in Table 2.

**Table 1:** Distribution of surgeons by region of origin (n=201).

Characteristics - n (%)	(n=201)
Region	
Midwest	13 (6.5)
Northeast	31 (15.4)
North	12 (6.0)
Southeast	129 (64.2)
South	16 (8.0)

**Table 2:** Number of surgeries performed per year (n=201).

Characteristics - n (%)	(n=201)
Number of surgeries	
0	49 (24.4)
<10	45 (22.4)
10 a 20	21 (10.4)
20 a 30	9 (4.5)
30 a 40	10 (5.0)
>40	67 (33.3)

The more frequent Orthopedics subspecialties among the orthopedic surgeons were: Traumatology (19.9%); Knee (19.4%); Hip (8.5%) and Shoulder and Elbow (7.0%). When asked whether they were visited by representatives of orthopedic implant companies, 51.5% reported receiving visits from representatives of those companies.

The prices indicated by orthopedic surgeons for various orthopedics materials and the suppliers of orthopedic implants are shown in Tables 3 and ^4^.

**Table 3:** Prices (R$) indicated as probable by orthopedists.

Materials	(n=201)
Total knee prosthesis	
mean (Standard deviation)	14281.09 (11811.20)
median	10000
minimum - maximum	1500.00 - 100000.00
Total hip prosthesis	
mean (Standard deviation)	16622.89 (13906.86)
median	14000
minimum - maximum	1000.00 - 100000.00
Shaver	
mean (Standard deviation)	2838.31 (4965.69)
median	1500
minimum - maximum	200.00 - 60000.00
Interference Screw	
mean (Standard deviation)	2359.45 (3277.99)
median	1500
minimum - maximum	50.00 - 30000.00
Radiofrequency	
mean (Standard deviation)	4252.49 (8982.63)
median	2000
minimum - maximum	100.00 - 100000.00
Locked Intramedullary Nail	
mean (Standard deviation)	9493.08 (56292.01)
median	4000
minimum - maximum	110.00 - 800150.00
Anchor	
mean (Standard deviation)	2752.14 (3256.61)
median	2000
minimum - maximum	100.00 - 25000.00

**Table 4:** Prices (R$) indicated by supplier companies of orthopedic materials.

Materials	
Total knee prosthesis	
mean	23390
minimum - maximum	18000 - 29820
Total hip prosthesis	
mean	27717
minimum - maximum	25000 - 32000
Shaver	
mean	800
minimum - maximum	500 - 1200
Interference Screw	
mean	1767
minimum - maximum	1000 - 2800
Radiofrequency	
mean	1933
minimum - maximum	1400 - 2400
Locked Intramedullary Nail	
mean	14977
minimum - maximum	11400 - 19080
Anchor	
mean	2433
minimum - maximum	1500 - 3800

The mean difference between the average prices expected by the surgeons and the average prices provided by the companies was 47.1%. This was a positive difference, i.e., the average prices given by most physicians were higher than the average commercial prices for the following materials: shaver (71.8%), anchor (11.6%), radiofrequency (54.5%) and interference screw (25.1%). As for the other materials (total knee prosthesis, total hip prosthesis and locked intramedullary nail of the tibia), the difference was negative, respectively, 63.8%, 66.7% and 36.6%. No significant differences were found between orthopedic specialists and other subspecialties regarding prices indicated for specific orthopedics materials. (Table 5)

**Table 5:** Prices of materials (R$) according to the orthopedists subspecialties.

Prices indicated as probable by orthopedists	Orthopedists	
Total knee prosthesis	Not knee specialist (n=162)	Knee specialist (n=39)
mean (Standard deviation)	14472,22 (12454,42)	13487,18 (8731,74)
median	10000	12000
minimum - maximum	1500,00 - 100000,00	1500,00 - 50000,00
p-value (Mann-Whitney test)	0,687	
Total hip prosthesis	Not hip specialist (n=184)	Hip specialist (n=17)
mean (Standard deviation)	16758,15 (14307,99)	15158,82 (8576,35)
median	14000	12000
minimum - maximum	1000,00 - 100000,00	3000,00 - 35000,00
p-value (Mann-Whitney test)	0,786	
Locked Intramedullary Nail	Not trauma specialist (n=161)	Trauma specialist (n=40)
mean (Standard deviation)	62854,74 (10142,61)	6878,75 (5777,46)
median	4000	5000
minimum - maximum	110,00 - 800150,00	850,00 - 25000,00
p-value (Mann-Whitney test)	0,059	

Significant differences were found between the orthopedists who receive visits from companies representatives and those who did not regarding prices indicated for shaver (p = 0.028) and radio frequency (p = 0.033). The orthopedic surgeons who received visits indicated lower prices for the same materials. (Table 6)

**Table 6:** Prices of Materials (R$) according to the visit of commercial representatives.

Materials	Receive visits from companies representatives	
	Yes (n=103)	No (n=97)
Total knee prosthesis		
mean (Standard deviation)	13087.38 (9145.19)	15438.14 (14060.37)
median	10000	10000
minimum - maximum	1500.00 - 50000.00	1500.00 - 100000.00
p-value (Mann-Whitney test)	0.58	
Total hip prosthesis		
mean (Standard deviation)	16249.51 (14435.62)	16675.26 (13028.82)
median	12000	15000
minimum - maximum	1500.00 - 100000.00	1000.00 - 80000.00
p-value (Mann-Whitney test)	0.625	
Shaver		
mean (Standard deviation)	2383.50 (3171.26)	3340.21 (6338.28)
median	1500	2000
minimum - maximum	200.00 - 20000.00	200.00 - 60000.00
p-value (Mann-Whitney test)	0.028	
Interference screw		
mean (Standard deviation)	2430.58 (3682.71)	2305.15 (2814.83)
median	1500	1500
minimum - maximum	200.00 - 30000.00	50.00 - 20000.00
p-value (Mann-Whitney test)	0.891	
Radiofrequency		
mean (Standard deviation)	3207.77 (5629.46)	5400.52 (11479.48)
median	1800	2000
minimum - maximum	100.00 - 40000.00	300.00 - 100000.00
p-value (Mann-Whitney test)	0.033	
Locked Intramedullary nail		
mean (Standard deviation)	4660.29 (3426.30)	6473.71 (6560.79)
median	4000	4000
minimum - maximum	110.00 - 20000.00	300.00 - 30000.00
p-value (Mann-Whitney test)	0.307	
Anchor		
mean (Standard deviation)	2402.91 (2739.52)	3148.25 (3710.63)
median	1800	2000
minimum - maximum	300.00 - 25000.00	100.00 - 25000.00
p-value (Mann-Whitney test)	0.18	

Significant correlation coefficients were found between the time of experience of orthopedic surgeons and prices indicated for the shaver (r = -0.30 p <0.001) and interference screw (r = -0.19 p = 0.007). The coefficients are negative, indicating that the higher experience time, the lower the price indicated for these materials. (Table 7)

**Table 7:** Analysis of correlation between material's prices (R$) and time of experience of the orthopedists.

Probable prices indicated by orthopedists (R$)	Spearmen correlation coefficient (r)	p-value
Total knee prosthesis	0.14	0.051
Total hip prosthesis	0.09	0.227
Shaver	-0.3	< 0.001
Interference screw	-0.19	0.007
Radiofrequency	-0.09	0.189
Locked intramedullary nail	-0.01	0.874
Anchor	-0.03	0.629

## DISCUSSION

The main result of this study is the low awareness of orthopedic surgeons on the prices of materials used in surgical procedures. This is a worrying result, as 60% of health-related costs are controlled by the doctors' decisions, although they receive little information and training on actions and strategies to reduce these costs.^9^

Streit et al.^4^ in a study that applied questionnaires to orthopedic surgeons, showed that the error in the estimate of orthopedics materials prices was 69%, and most of these errors (67%) underestimated the prices ​​of orthopedics materials. Another study on orthopedic implants costs showed that only 21% of doctors estimated correctly the values ​​of the materials provided by empresas.5 In this study the difference between the prices estimated by doctors and the actual price provided by companies was 47%; there was both underestimation and overestimation of prices by the surgeons in, respectively, 3 and 4 types of orthopedics materials.

Burns et al.^2^ showed a close and long term relationship between surgeons and implant manufacturers. However, only a small part of the orthopedic surgeons received financial payments from supplier companies.^2^ ^,^ ^10^ In Brazil, in our surgeons' sample, more than half (51.5%) reported receiving visits from representatives of orthopedic implant companies. However, differences were only found between the orthopedists who received visits from representatives of companies and those who did not. Considering shaver and radiofrequency, the orthopedic surgeons visited by commercial representatives underestimated their prices.

Okike et al.^5^ showed that medical residents thought they had worse knowledge on the costs of orthopedic implants that more experienced doctors. This study found differences between the experience time of orthopedists and prices listed for some materials; the longer the surgeons' experience time, the lower the price indicated for some materials. However, no differences were found regarding the subspecialty referred by the orthopedic surgeons and the prices indicated for orthopedics materials specific for this particular subspecialty.

Despite the increasing costs in health care, a study published by the American Society of Orthopaedic Surgeons showed that most surgeons did not consider themselves responsible for containing health costs.^11^ We do know, however, that physicians have a key role in this economic process and in reducing health costs. A previous study showed that 85% of patients proved willing to pay additional amounts for best quality materials indicated by doctors even whether they were not covered by health insurance plans.^12^ While cost containment is critical to the viability and maintenance of health systems, care should be taken to ensure that cost-effectiveness does not increase the number of complications and compromise the patient's outcomes.^11^ ^,^ ^13^

Closer cooperation between the various health stakeholders such as hospitals, doctors, health plans and orthopedic implant companies is required to achieve the goal of significantly reducing implant costs, maintaining the quality of services provided to patients.^11^

## CONCLUSION

The knowledge of Brazilian orthopedic surgeons on the costs of orthopedic implants is feeble. Cost reduction of orthopedics materials depends on a more effective communication and interaction between doctors, hospitals and supplier companies with more solid orientation and awareness programs for physicians about their importance in this scenario.
